# Identification and functional characteristics of a novel splicing heterozygote variant of *COL2A1* associated with Stickler syndrome type I

**DOI:** 10.3389/fgene.2024.1308737

**Published:** 2024-07-10

**Authors:** Yujing Gong, Weijian Zhu, Mianmian Zhu, Dan Chen, Sunke Wu, Sisi Hu, Yi Luo, Yiyi Jiang, Ting Zhu, Dan Wang

**Affiliations:** ^1^ Department of Pediatrics, The First Affiliated Hospital of Wenzhou Medical University, Wenzhou, Zhejiang, China; ^2^ Central Laboratory, The First Affiliated Hospital of Wenzhou Medical University, Wenzhou, Zhejiang, China; ^3^ Department of Pediatrics, Yongjia People’s Hospital, Wenzhou, Zhejiang, China; ^4^ Department of Pediatrics, Taizhou Woman and Children’s Hospital, Taizhou, Zhejiang, China; ^5^ Department of Radiography, The First Affiliated Hospital of Wenzhou Medical University, Wenzhou, Zhejiang, China

**Keywords:** Stickler syndrome, functional analysis, COL2A1 gene, intron heterozygote variant, type II collagenopathies

## Abstract

**Background:**

Stickler syndrome type I (STL1) is an autosomal dominant disorder characterized by ocular, auditory, orofacial, and skeletal anomalies. The main causes of STL1 are variants in the *COL2A1* gene, which encodes a type II collagen precursor protein. The specific focus of this study was on a newborn from China diagnosed with STL1, with the aim of providing novel insights into the effects of a newly identified intronic variant in the *COL2A1* gene on pre-mRNA splicing.

**Methods:**

Trio whole exome sequencing was used to identify the causative variant in the family. The identified variant was validated using Sanger sequencing. Bioinformatics programs were used to predict the pathogenicity of the candidate variant. Additionally, an *in vitro* minigene assay was used to investigate the effects of the identified variant on RNA splicing.

**Results:**

The proband with STL1 had a novel heterozygous splicing variant in the intron nine acceptor donor site of *COL2A1* (c.655-2A>G). This splice junction variant resulted in aberrant *COL2A1* mRNA splicing, leading to the skipping of exon 10 and the production of a shorter protein that may lack the last 18 native amino acids.

**Conclusion:**

The c.655-2A>G variant in the *COL2A1* gene leads to STL1 through abnormal splicing. By expanding the spectrum of variants in the *COL2A1* gene, this finding improves the clinical understanding of STL1 and provides guidance for early diagnosis and disease counseling.

## 1 Introduction

Stickler syndrome is a collagen connective tissue disease characterized by ocular, auditory, orofacial, and skeletal anomalies, with dominant inheritance being common and recessive inheritance being rare (Snead and Yates, 1999). The incidence rate of Stickler syndrome in neonates is approximately 1 in 7,500–9,000 (Hoornaert et al., 2010). Based on locus heterogeneity, autosomal dominant hereditary Stickler syndrome is divided into three types: I, II, and III. The most prevalent form, Stickler syndrome type I (STL1, MIM #108300), is predominantly caused by defects in the *COL2A1* gene (Faber et al., 2000). STL2 (MIM #604841) is associated with defects in *COL11A1* and patients have exhibited the “beaded” type 2 vitreous phenotype (Poulson et al., 2004), (Wilkin et al., 1999). Type III, also known as non-ocular Stickler syndrome or otospondylomegaepiphyseal dysplasia, is caused by pathogenic variants in *COL11A2* (Annunen et al., 1999). The symptoms and signs of patients diagnosed with STL exhibit wide variations. STL1 may present with a wide range of findings, including cleft palate, small jaw, hearing loss, or early-onset osteoarthritis (Stickler et al., 2001). STL1 is most commonly associated with ocular complications, but inner ear, skeletal/joint, and craniofacial structures are often involved. The clinical characteristics of STL1 include typical vitreous (membranous) lesions accompanied by retinal detachment; facial abnormalities, such as cleft palate, glossophthalmos, and retropalatal deformity; relatively mild corresponding osteophytes; and early-onset arthropathy. The phenotype of STL1 constantly evolves throughout life, making diagnosis challenging, particularly in sporadic cases. Therefore, it is crucial to diagnose this disease by combining phenotypic and genetic analyses (Boothe et al., 2020).

The *COL2A1* gene maps on to 12q13.11-q13.2, contains 54 exons, and encodes the type II collagen precursor protein (Nishimura et al., 2005). Alterations in the amino acid arrangement of type II collagen due to *COL2A1* variants can affect the structural stability and function of the protein helix, leading to type II collagenopathies (MIM #120140). (Annunen et al., 1999; Wilkin et al., 1999; Yamamoto et al., 2020). Type II collagenopathies can be broadly classified into five categories: lethal diseases, types mainly affecting spinal deformity, types involving long bones and joints, types primarily associated with ocular manifestations, and unusual types. Although STL1 belongs to the type mainly involving ocular manifestations, it is also characterized by skeletal dysplasia (Zhang et al., 2020). The most frequent manifestation of this disease during the neonatal period is a cleft palate (Hanspal et al., 2012). STL1 is the mildest type of collagen II disease (Barat-Houari et al., 2016; Zhang et al., 2020).

In this study, we present the case of a Chinese infant whose clinical manifestations aligned with those of patients with Stickler syndrome. Through whole exome sequencing, a rare and previously undescribed intron variant of *COL2A1* (c.655-2A>G) was identified, broadening. The variant spectrum of *COL2A1.* Furthermore, we performed *in silico* analysis to investigate the possible molecular pathogenesis of Stickler syndrome in this patient.

## 2 Materials and methods

### 2.1 Participants

The proband and parents were enrolled at the First Affiliated Hospital of Wenzhou Medical University, where the proband was hospitalized because of a palatal deformity discovered during a post-birth physical examination. Relevant clinical records, including physical, routine blood, ultrasound, and oral computed tomography (CT) examination findings, were collected. Written consent was obtained from the parents of the newborn proband before commencing the study, which was approved by the Ethics Committee of the First Affiliated Hospital of Wenzhou Medical University.

### 2.2 Trio whole exome sequencing and sanger sequencing

Genomic DNA from blood samples (1–2 mL) of the proband and their family members was used in this assay. The DNA was first fragmented, and libraries were prepared; subsequently, the DNA in the all exons and the adjacent shear region were captured and enriched using the Roche KAPA HyperExome chip (KAPA Biosystems, Boston, MA, United States of America), and the MGISEQ-2000 sequencing platform (MGI Tech Co., Ltd., Shenzhen, Guangdong, China) was used for variant detection. The quality control index of the sequencing data was as follows: the average sequencing depth of the target region was ≥180X, and the percentage of loci with an average depth >20X in the target region was >95%. Sequenced fragments were mapped to the human reference genome (UCSC hg19) using BWA aligner (Burrows-Wheeler alignment tool, version 0.7.15) to remove duplicates. Base mass value correction for SNV, INDEL, and genotype detection was performed using GATK. ExomeDepth was used to detect copy number variation at the exon level. The main reference databases used in this study included population (ClinVar, ESP6500, and gnomAD) and clinical databases (Online Mendelian Inheritance in Man, Rare Disease Data Center [RDDC], GeneReviews, Orphanet, and Genetic Home Reference). According to the guidelines of the American College of Medical Genetics and Genomics, the pathogenicity of variants is divided into five categories. This is used to determine which category the variant is judged to be in, and further reference is provided for subsequent research. The suspected mutations were subsequently verified using Sanger sequencing (primer sequences: GTC​AGA​GTT​CCT​CCA​CAG​CTA​G and CCC​TCA​TTT​TCT​GTT​CCG​ATG​C) in the studied family. Sanger sequencing was performed using an ABI 3730xl DNA Analyzer (Applied Biosystems, Carlsbad, CA, United States of America).

### 2.3 *In silico* assay

The RDDC database (https://rddc.tsinghua-gd.org/) and varSEAK (https://varseak.bio/) were used to analyze the possible effects of the mutation. SpliceAI Lookup (https://spliceailookup.broadinstitute.org/#) was used for the mutation evaluation. The three-dimensional (3D) structure of the protein template after introducing mutations was predicted using PyMOL. The template protein structure (XP_016874318.1) was obtained from the National Center for Biotechnology Information protein database (https://www.ncbi.nlm.nih.gov/). The prediction method followed the standard procedures.

### 2.4 Minigene constructs and mutagenesis

An *in vitro* minigene assay was performed to examine the target gene regions covering *COL2A1* exons 10–11, intron 9, and intron 10, which were amplified from the gDNA of the proband. The wild-type *COL2A1* gene fragment was obtained by nested polymerase chain reaction (PCR) using genomic DNA as the template and *COL2A1*-7948-F/*COL2A1*-9565-R and *COL2A1*-8186-F/*COL2A1*-996-R (Table 1) as primers. Three pairs of primers (pcDNA3.1-*COL2A1*-KpnI-F/*COL2A1*-MUT-R, *COL2A1*-MUT-F/pcDNA3.1-*COL2A1*-EcoRI-R, and pcDNA3.1-*COL2A1*-KpnI-F/pcDNA3.1-*COL2A1*-EcoRI-R) were developed to amplify the heterozygous c.655-2A>G mutation site from the product of nested PCR via seamless cloning (Vazyme Biotech Co., Ltd., Nanjing, China). Subsequently, the amplified DNA products were recombined and cloned into two digestion sites (HIKpnI/EcoRI) of the pcDNA3.1 vector (Hitrobio Biotechnology Co., Ltd., Beijing, China). In addition, the recombinant plasmids pcDNA3.1-*COL2A1*-t (wild-type) and pcDNA3.1-*COL2A1*-mut (c.655-2A>G) were validated using Sanger sequencing.

**TABLE 1:** Primer sequences used to analyze the variant of the *COL2A1* gene and vector pcDNA3.1.

Primer name	Primer sequence (5` - 3′)
COL2A1-7948-F	tgc​cac​ttt​taa​tga​tgc​gct​g
COL2A1-8186-F	gaa​agc​aag​gcc​agc​ttt​tct​g
COL2A1-9565-R	ctc​tcc​tag​gtt​ctg​ctg​act​gt
COL2A1-9296-R	tcc​ctt​cct​ggg​gct​aat​gat​g
pcMINI-C-COL2A1-KpnI-F	ggt​aGG​TAC​Cta​tct​gca​att​ctt​ttt​gcc
pcMINI-C-COL2A1-EcoRI-R	TGC​AGA​ATT​CAT​CAT​CAC​CAG​GCT​TTC​CAG
pcDNA3.1-COL2A1-KpnI-F	GCT​TGG​TAC​CAT​GGG​CCC​CAT​GGG​ACC​TCG​AGG
pcDNA3.1-COL2A1-EcoRI-R	TGC​AGA​ATT​CAT​CAT​CAC​CAG​GCT​TTC​CAG
COL2A1-MUT-F	tca​ttt​tac​ttt​ttg​gGG​GCC​TCA​AGG​ATT
COL2A1-MUT-R	AAT​CCT​TGA​GGC​CCc​Caa​aaa​gta​aaa​tga
pcDNA3.1-F	CTA​GAG​AAC​CCA​CTG​CTT​AC
pcDNA3.1-R	GCA​CCT​TCC​AGG​GTC​AAG​GA

To confirm the conclusions drawn in this study, the assay was repeated using the same experimental procedure with another pcMINI-N vector containing the universal Exon A-intron A-MCS sequence. The cells were transfected, and the ExonA-Exon10-Exon11 splicing pattern was observed to determine whether it was abnormal, as previously described.

### 2.5 Cell culture and transfection

Human embryonic kidney (HEK293T) and HeLa cell lines (Cell Resource Center of the Chinese Academy of Medical Science, Beijing, China) were maintained at 37°C in a humidified environment using 5% CO_2_ in Dulbecco’s modified Eagle medium supplemented with 10% fetal bovine serum and 1% penicillin-streptomycin. *COL2A1*-wt and variant cDNA were inserted into the pcDNA3.1 vector according to the manufacturer’s instructions, and HEK293T and HeLa cells were transfected using Lipo3000 transfection reagent (GlpBio Technology Inc., Montclair, CA, United States of America). The transfected cells were incubated for 48 h prior to RNA extraction.

### 2.6 RNA extraction, polymerase chain reaction, and sequencing

Total RNA from HEK293T and HeLa cells transfected with small gene plasmids was extracted using the GenElute Mammalian Total RNA Kit (Sigma-Aldrich) according to the manufacturer’s recommendations. DNA was degraded on the columns using DNA enzyme I (Qiagen, Valencia, CA, United States of America). The extracted total RNA was reversely transcribed to cDNA, and a primer was used for the retropolymerase chain reaction of pcDNA3.1-F/pcDNA3.1-R (Table 1). Next, the cDNA products were detected using 1% agarose gel electrophoresis and further validated via Sanger sequencing. The lengths of the wild-type cDNA products were 396 bp in the pcDNA3.1-*COL2A1* vector and 537 bp in the pcMINI-N-*COL2A1* vector.

## 3 Results

### 3.1 Clinical data

**FIGURE 1:**
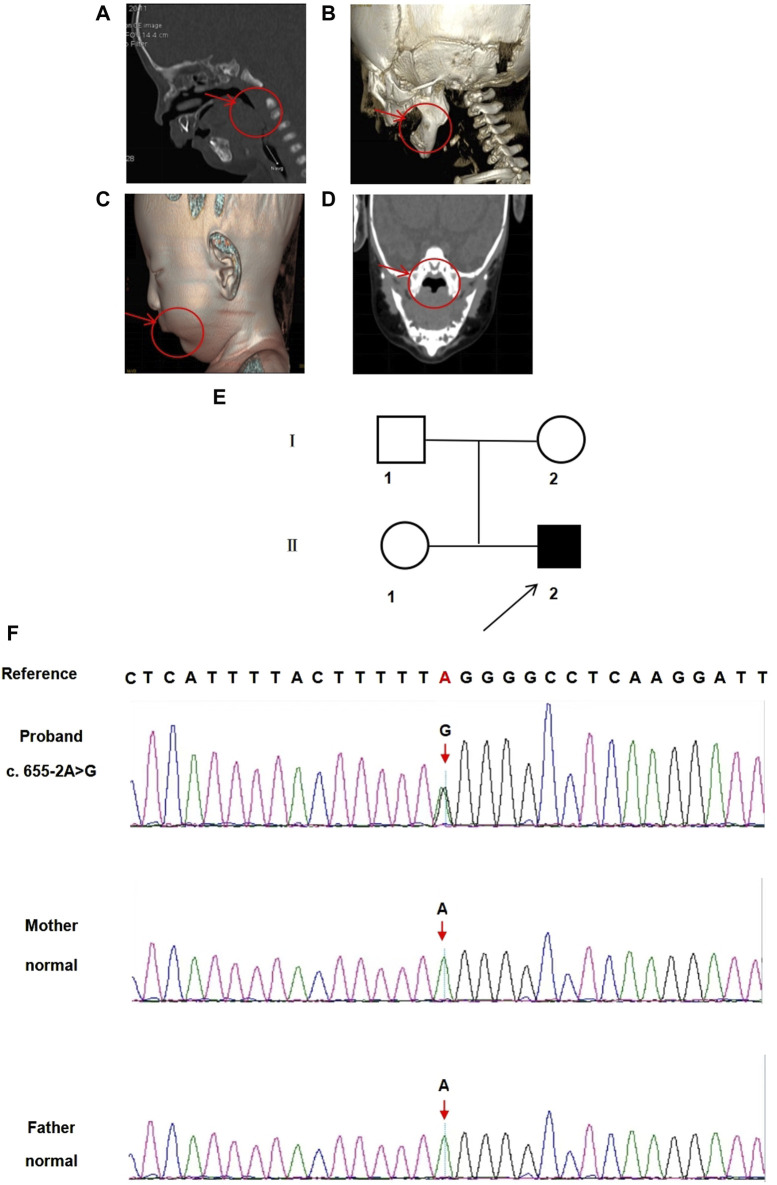
CT images and 3D reconstructions were conducted for this infant. **(A)** The arrow indicates back tongue root in the CT sagittal axial view. **(B)** The arrow indicates small jaw in the 3D reconstructions sagittal axial view. **(C)** The arrow indicates small jaw in the 3D reconstructed sagittal view. **(D)** The arrow indicates a cleft palate in the CT coronal view. **(E)** Pedigree of the family. □ represents the normal male; ○ represents the normal female; ■ represents the affected male. Arrow indicates the proband. **(F)** The sequencing result of the family. Sanger sequencing showing *COL2A1* **(C)**.655-2A>G variant. The arrow symbol indicates the mutation site.

The male proband, born to a non-consanguineous 43-year-old mother and a 56-year-old father, was the second child in the family. The mother’s pregnancy was complicated because of connective tissue disease and gestational diabetes mellitus. However, there was no reported family history of genetic diseases. Pregnancy care revealed no exposure to teratogenic or infectious agents. He was delivered at the 38th week of gestation via spontaneous vaginal delivery, weighing 2,800 g (10th–25th percentile) and measuring 50 cm in length (50th–75th percentile). His Apgar scores were 9 and 10 at 1 and 5 min, respectively. Physical examination at birth revealed a small jaw, back tongue root, cleft palate, and intact hard palatal mucosa. Shortly after birth, the proband was hospitalized because of dyspnea and feeding difficulties. CT findings and 3D reconstructions revealed mandibular retraction, glossoptosis, airway stenosis, and incomplete cleft palate (Figures 1A–D). Additionally, abdominal ultrasonography revealed right renal pelvic separation, and an echocardiogram indicated a patent foramen ovale. Additionally, the result of universal newborn hearing screening was positive for hearing loss. Blood biochemical test results for total protein, albumin, alanine aminotransferase, leukocytes, hemoglobin, red blood cells, and platelet counts were all within normal limits. His cleft palate was surgically repaired 8 days after birth. Up to the age of 18 months, he exhibited normal motor and language development without experiencing symptoms of retinal detachment.

### 3.2 Variant detection

A novel heterozygous point variant in the intron 9 (c.655-2A>G, NM_001844.4) splice donor site of *COL2A1* (NC_000012.12) was identified in the proband (II-2, Figure 1E) using trio exome sequencing and confirmed by PCR-based Sanger sequencing (Figure 1F). DNA analyses of the parents (I-1 and I-2) and sister (II-1) were normal, indicating that the variant was *de novo* in the proband.

### 3.3 *In silico* assay

**FIGURE 2:**
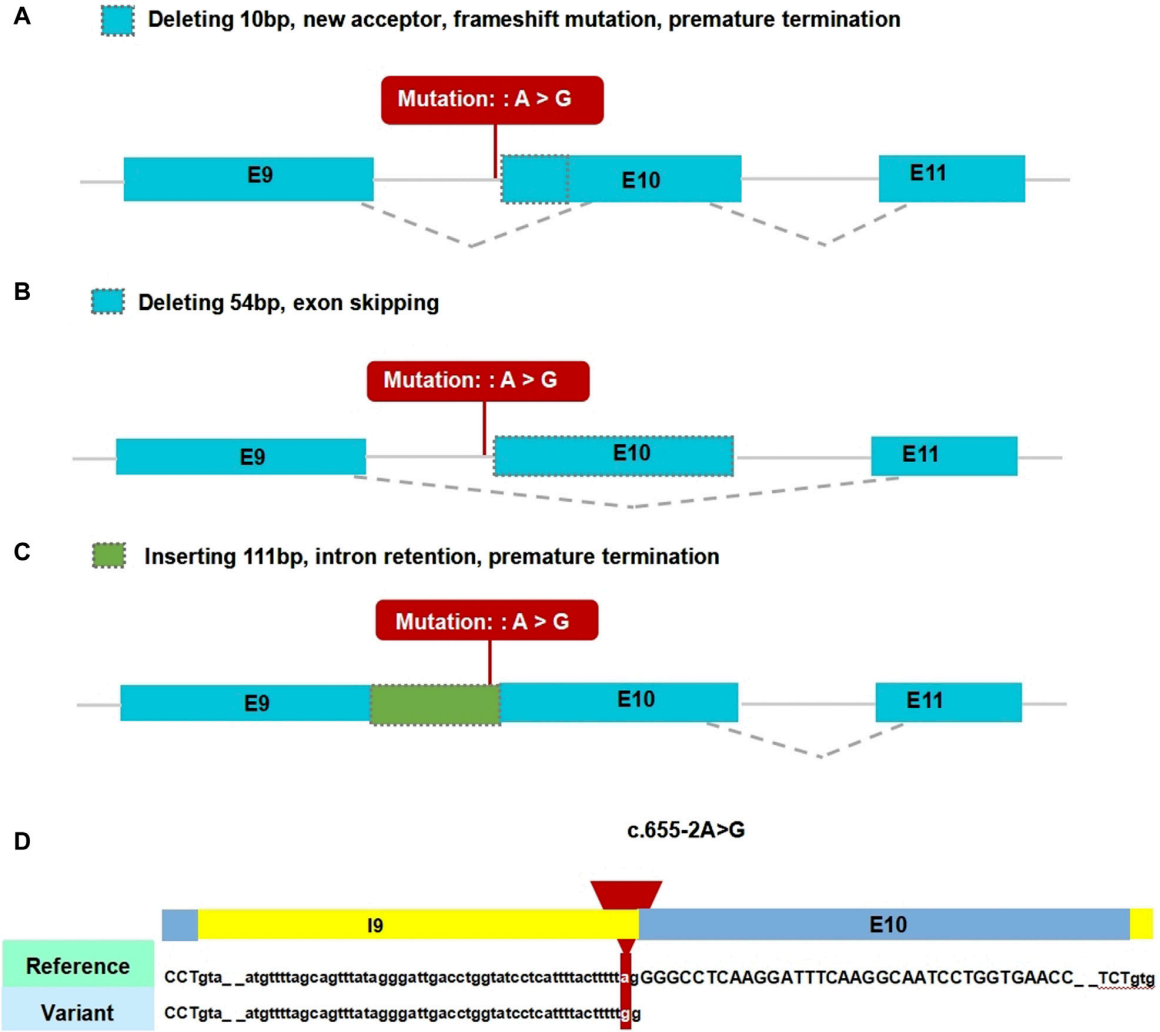
Results of *in silico* assay. A-C. Three patterns of RNA splicing prediction. **(A)** Pattern one: Deleting 10 bp, new acceptor, frameshift mutation, premature termination. **(B)** Pattern two: Deleting 54 bp, exon skipping. **(C)** Inserting 111 bp, intron retention, premature termination. The minigene results showed that our variant of interest was consistent with pattern two. **(D)** Result of the prediction in varSEAK online consists of these *in silico* assays by Human Splicing Finder (HSF). E9: exon 9; E10: exon 10; E11: exon 11.

In the gnomAD database, the *COL2A1* variant (c.655-2A>G) is not included and has not been reported previously, indicating that this is a novel variant (Table 2). Functional prediction of the *COL2A1* variant was performed using the RDDC platform. The analysis indicated that this variant potentially had pathogenic effects, as suggested by the three different predicted patterns of RNA splicing (Figures 2A–C). For c.655-2A>G, the splice AI algorithm returned a high value (Δ Score = 1.00). This value, which was above the high precision threshold (Δ Score ≥0.8), was used to detect higher sensitivity of splice change variants (Table 3). These results were validated using varSEAK (Figure 2D). Mutations in the 3′acceptor splice site could lead to exon skipping (Table 4). Our analyses suggested that the detected *COL2A1* variant (c.655-2A>G) is a novel variant that affects mRNA splicing.

**TABLE 2:** Intronic variations of *COL2A1* previously reported in gnomAD database.

Variant.ID.	HGVS.Consequence
12-48389627-G-I	c.654 + 37C>A
12-48389626-C-T	c.654 + 32G>A
12-48389622-G-A	c.654 + 36C>T
12-48389621-C-T	c.654 + 37G>A
12-48389620-G-C	c.654 + 38C>G
12-48389619-T-C	c.654 + 39A>G
12-48389618-C-T	c.654 + 40G>A
12-48389609-G-A	c.654 + 49C>T
12-48389601-T-C	c.655-55A>G
12-48389597-A-T	c.655-51T>A

**TABLE 3:** Splicing mutation evaluation by SpliceAI.

Acceptor loss	Donor loss	Acceptor Gain	Donor Gain
1.00	0.00	0.69	0.00
-2bp	—	−12bp	—

**TABLE 4:** Assessing splicing impact with varSEAK.

Acceptor loss	Splicing effect	Class of splicing effect
Predictions 5′Donor Splice Site	No splicing effect	1
Predictions 3′Acceptor Splice Site	Exon skipping	5

### 3.4 Minigene assay

**FIGURE 3:**
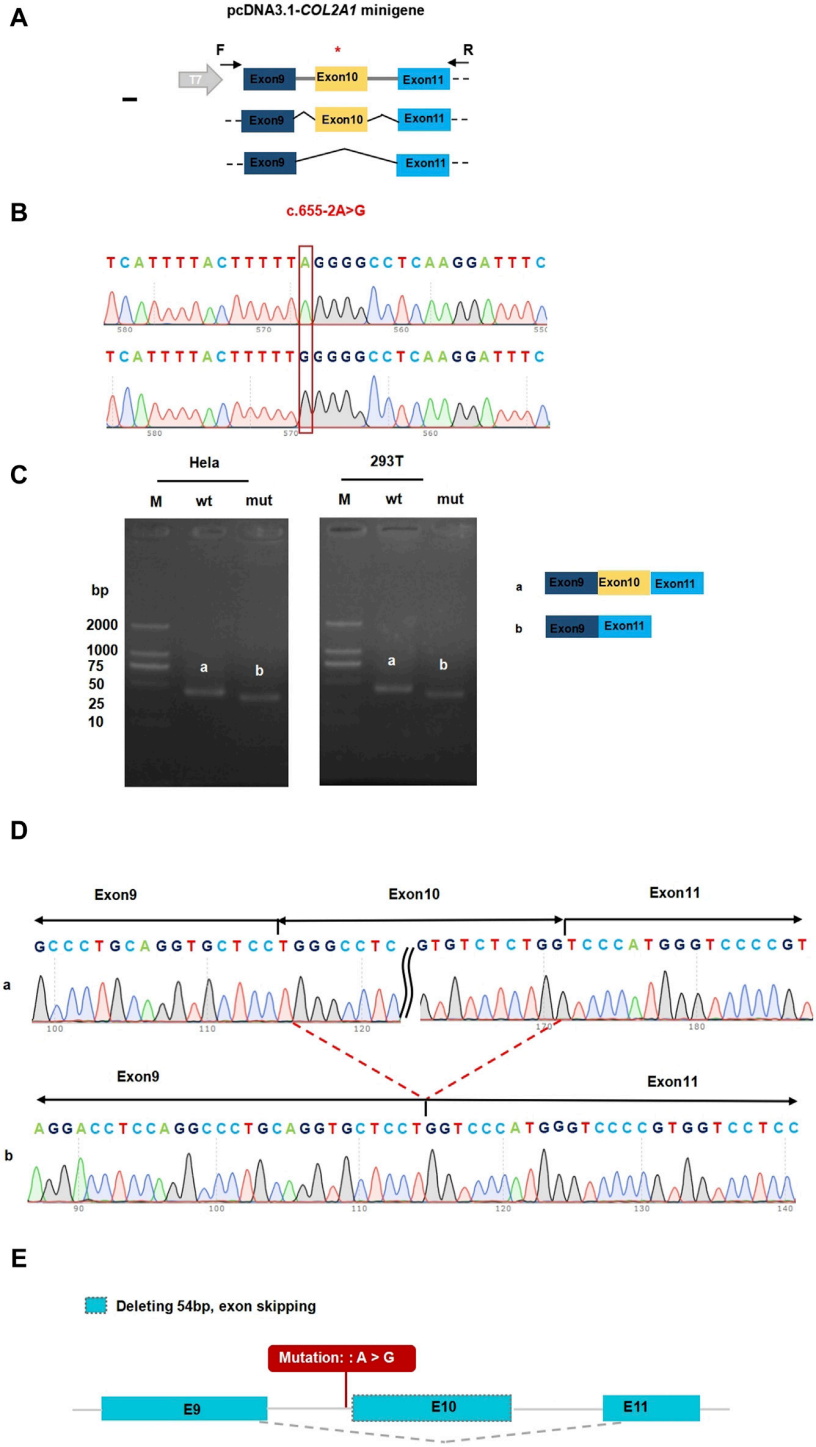
Functional analysis of the *COL2A1* variant effect on mRNA splicing. **(A)** Construction strategy of pcDNA3.1 minigene vector, * representative of the variant site. **(B)** The sequencing of wildtype *COL2A1*gene vector and **(C)**.655-2A>G *COL2A1* variant gene vector. **(C)** Gel electrophoresis of reverse transcription polymerase chain reaction products displayed a single band a (estimated 396 bp) from the wild type (wt) and a smaller band b (estimated 342 bp) in the mutant type (mut). **(D)** Illustration of the sequencing of band a (wild type in HEK293T and Hela cells) and band b (variant c.655-2A>G in HEK293T and Hela cells) products lead to a shorter transcript with deletion of exon 10 including 54 bp. **(E)** Accurate result of the prediction *in silico* assay: Deleting 54 bp, exon skipping.

*In vitro* transcriptional assays were performed to analyze the effect of intron donor locus variation on *COL2A1* mRNA splicing. The pcDNA3.1-*COL2A1* small gene was 665 bp long and covered DNA regions, including exon 9 (45 bp), intron 9 (111 bp), exon 10 (54 bp), intron 10 (401 bp), and exon 11 (54 bp, Figures 3A, B). A full-length reverse transcription PCR product of 396 bp, consisting of a partial plasmid sequence of 243 bp and a target gene of 153 bp, was detected in HEK293T and HeLa cells transfected with the pcDNA3.1-*COL2A1* minigene (Figure 3C). Sanger sequencing confirmed that the 396 bp band corresponded to normal *COL2A1* mRNA, in which exons 9–10–11 were spliced and introns 9–10 were removed between them (Figure 3D). The pcDNA3.1-*COL2A1*-mut minigene containing the c.655-2A>G variant was transfected into the HeLa and HEK293T cell lines. In contrast, normal *COL2A1* mRNA was not detected in the mutant (MUT) swim channel, except for a smaller band (342 bp, Figure 3C). In the MUT lanes, an aberrantly spliced transcript of *COL2A1* was observed as a short band of 342 bp, which included only exons nine and 11, with exon 10 being completely skipped. A Sanger DNA chromatogram of the misspelled transcript of *COL2A1* is shown in Figure 3D. The results from the assay conducted in the pcMINI-N vector were consistent with previous findings, showing the skipping of exon 10. Translational analysis of the full-length sequence of the misspliced transcript identified by small gene analysis showed that this variant induced complete skipping of exon 10, including 54 bp nucleotides (c.655_708del), resulting in a deletion of 18aa within the shorter protein (p.[Gln221_Pro238del]). These results were consistent with the predictions of *in silico* assays, and the RNA splicing pattern was consistent with the predictions of splicing pattern II (Figure 3E).

### 3.5 Three-dimensional structure of the protein

**FIGURE 4:**
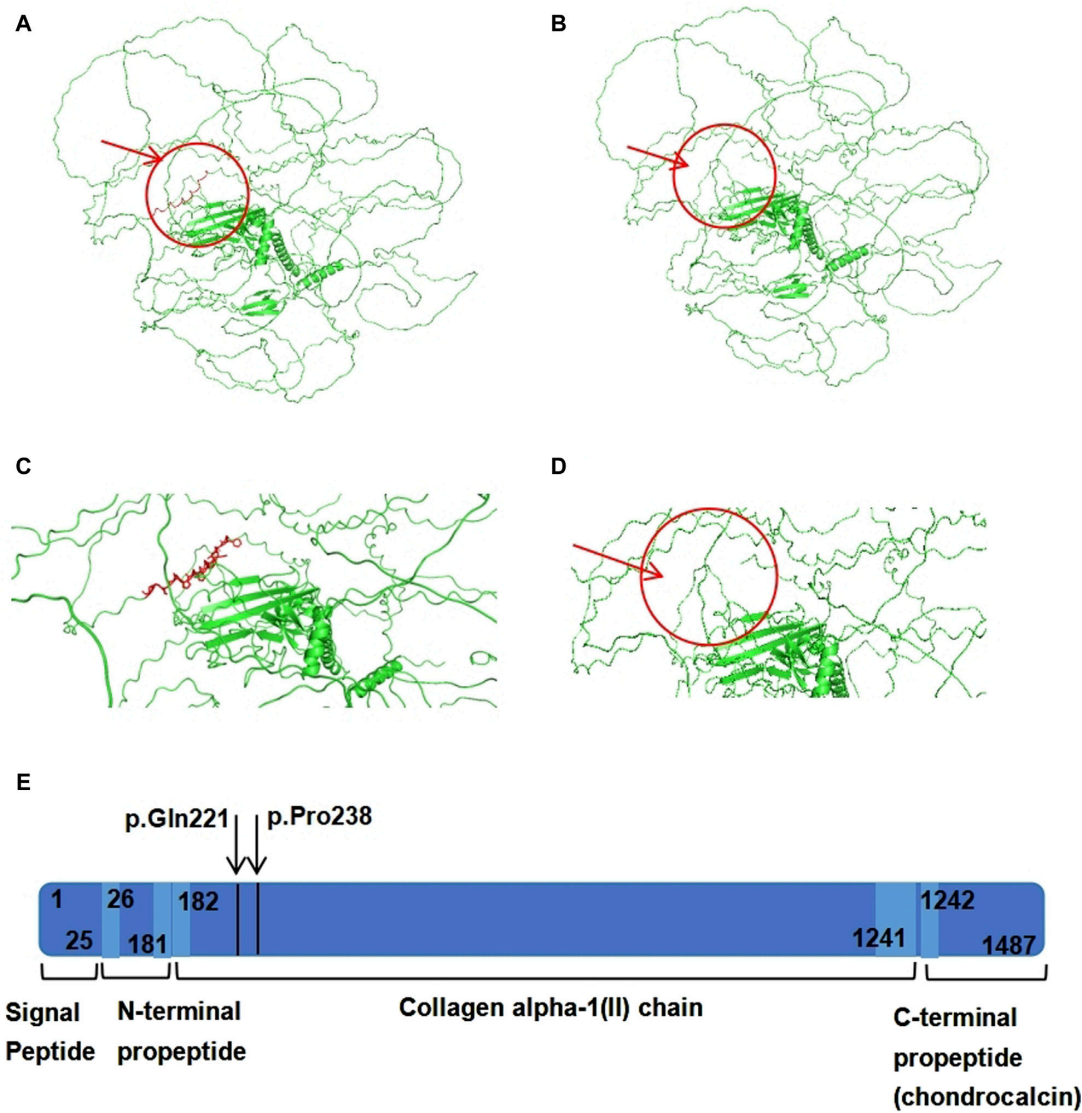
The domain and three-dimensional structures of the wildtype and variant *COL2A1* proteins. **(A)** The 3D structure of wild-type *COL2A1* protein. **(B)** The 3D structure of variant *COL2A1* protein. **(C)** Localized amplification of the 18 amino acids (p.[Gln221_Pro238del]). **(D)** 3D structure of variant *COL2A1* protein. The arrow indicates deletion of 18 amino acids. **(E)** Schematic representation of *COL2A1* protein domains, regions and peptides. The arrow indicates deletion of 18 amino acids.

The 3D structure prediction of the variant protein was analyzed using the PyMOL software. Schematic diagrams of the primary and variant models of the *COL2A1* protein are shown in Figures 4A–D. Compared with the wild-type sequence, the variant protein had an altered amino acid sequence, resulting in a shorter protein variant with a loss of 18 native amino acids (p.[Gln221_Pro238del]). The loss of 18 native amino acids are predicted to not affect any functional domains (Figure 4E).

## 4 Discussion

In this study, we described the case of a male infant with cleft palate, small lower jaw, glossoptosis, retrognathia, and abnormal hearing. The infant was found to have a novel heterozygous variant, c.655-2A>G in *COL2A1*, and this had not been previously reported. This splice junction variant led to aberrant *COL2A1* mRNA splicing, resulting in the formation of an alternative transcript that produces a shorter *COL2A1* protein. The male infant was diagnosed at birth with Pierre Robin sequence (small lower jaw, glossoptosis, cleft palate and airway problems) and did not pass the newborn hearing screening test. Based on the clinical manifestations and imaging results, the proband was diagnosed with STL1.

The *COL2A1* gene is located on 12q13.11 and contains 54 exons. The gene’s helix region, encoded by codons 201–1,214, consists of a core repeat of three residues, all beginning with a glycine (Gly-X-Y), where “X” typically denotes proline and “Y” represents hydroxyproline residues (Barat-Houari et al., 2016). The coding sequence of this gene is highly conserved among different species. (Dale and Topczewski, 2011). The *COL2A1* gene encodes the α-1 chain of type II procollagen. Type II collagen is a homotrimer of three α-1(II) procollagen chains (Terhal et al., 2012; Nenna et al., 2019). Type II collagen is the primary component of the extracellular matrix in transparent cartilage, intervertebral disc nucleus, inner ear structures, and vitreous body. It acts as an autocrine factor for proliferation and differentiation through various downstream effectors and inhibits chondrocyte apoptosis via the suppression of BMP-SMAD1 pathway activity (Lian et al., 2019), thereby playing a crucial role in cartilage formation and growth, especially in relation to bone development within the cartilage. Various pathogenic or potentially pathogenic mutations in the *COL2A1* gene have been reported, including missense and loss-of-function mutations (Hoornaert et al., 2010). Most of these lead to the introduction of a premature stop codon and nonsense-mediated decay, resulting in the production of abnormal type II collagen fibers (Stickler et al., 2001; Hoornaert et al., 2010). This study focused on a novel splice-site receptor variant of *COL2A1* in intron 10, emphasizing the pathogenicity of intronic variants. The two nucleotides directly adjacent to the intron–exon junction in the splicing region are highly conserved, with the bases at the 5'(donor site) and 3'(acceptor site) ends of the intron almost always being GT and AG, respectively, according to the GT-AG rule. In the mammalian genome, the probability of a splicing site conforming to the classical GT-AG combination is 98.71% (Mercuri et al., 2005). Consequently, a splice site receptor variant in this conserved region is likely to be pathogenic. Our analysis, using various algorithms and minigene assays, demonstrated that the novel variant disrupted splicing receptor function, causing complete skipping of exon 10 and resulting in the deletion of 18 amino acids within the shorter protein (Figures 4A–D). Therefore, we believe that this novel splice-site variant is pathogenic.

Pathogenic variants in the *COL2A1* gene lead to a series of diseases collectively referred to as type II collagenopathies, which are characterized by skeletal dysplasia (Stickler et al., 2001; Barat-Houari et al., 2016). Variations in the *COL2A1* gene can lead to abnormal phenotypes in the skeletal, craniofacial, auditory, and visual systems. Based on severity, the clinical phenotypes of *COL2A1* gene variations can be classified into lethal, severe, and mild types. Stickler syndrome is classified as mild type (Zhang et al., 2020). STL1, also known as hereditary progressive osteo-ophthalmic disease, is a type II collagenopathy phenotype associated with retinal detachment, facial abnormalities, cleft palate, and mild abnormalities in the development of spinal bone growth centers (Čopíková et al., 2020). The phenotypes of STL1 are diverse. Physical examination of the proband revealed a small jaw, posterior lingual base, and cleft palate. He was hospitalized shortly after birth because of breathing and feeding difficulties. The patient was diagnosed at birth using the Pierre Robin sequence. Similar to the proband, characteristics of midfacial hypoplasia were evident in the infant. Infants with the Pierre Robin sequence experience difficulties in breathing and feeding during early infancy exclusively (Stickler et al., 2001). Variations affecting splicing can lead to nonsense-mediated decay or produce an in-frame protein with a small deletion (Bruni et al., 2021). It is generally believed that the production of an in-frame protein with a small deletion results in severe phenotypes. A recent study showed that variants with deletions of ≤18 amino acids were associated with less severe phenotypes (Bruni et al., 2021). In this case, the detected a splice site variant that results site variant resulted in an internal deletion of 18 amino acids but with a milder phenotype. This suggests that the STL1 phenotype may be related to the size of the in-frame deletion. It has been suggested that midfacial hypoplasia in neonates should be considered during STL1. Based on the clinical presentation of the patient and the results of trio whole exome sequencing and an *in vitro* minigene assay, we consider the patient to be diagnosed with Stickler syndrome type I.

In conclusion, we identified a *de novo* heterozygous intronic variant, c.655-2A>G, in *COL2A1* and verified its pathogenicity using an *in vitro* in *COL2A1* using whole exome sequencing and an *in vitro* minigene assay. Abnormalities in intronic splicing sites are essential for predicting splicing variants, and in this case, they lead to aberrant *COL2A1* mRNA splicing, resulting in the complete skipping of exon 10 (c.655_708del) and deletion of 18 amino acids (p.[Gln221_Pro238del]). This suggests that the phenotypes of STL1 may be associated with the size of in-frame deletion. Furthermore, our findings expand the spectrum of *COL2A1* variants. Therefore, genetic testing should be performed on any individual suspected of having STL1, as it plays a crucial role in guiding patient management (ACMG Board of Directors, 2015).

## Data Availability

The original contributions presented in the study are publicly available. This data can be found here: https://www.ncbi.nlm.nih.gov/clinvar/variation/2627375/?oq=SUB13902533&amp;m=NM_001844.5(COL2A1):c.655-2A%3EG.
